# Properties and Characterization Techniques of Graphene Modified Asphalt Binders

**DOI:** 10.3390/nano13050955

**Published:** 2023-03-06

**Authors:** Rodrigo Polo-Mendoza, Tatiana Navarro-Donado, Daniela Ortega-Martinez, Emilio Turbay, Gilberto Martinez-Arguelles, Rita Peñabaena-Niebles

**Affiliations:** 1 Faculty of Science, Charles University, 128 00 Prague, Czech Republic; 2 Department of Civil & Environmental Engineering, Universidad del Norte, Barranquilla 081001, Colombia; 3 School of Civil and Environmental Engineering, Technische Universität Dresden, 01069 Dresden, Germany; 4 Department of Industrial Engineering, Universidad del Norte, Barranquilla 081001, Colombia

**Keywords:** asphalt binder, graphene, graphene-like materials, modifying agents, nanomaterials

## Abstract

Graphene is a carbon-based nanomaterial used in various industries to improve the performance of hundreds of materials. For instance, graphene-like materials have been employed as asphalt binder modifying agents in pavement engineering. In the literature, it has been reported that (in comparison to an unmodified binder) the Graphene Modified Asphalt Binders (GMABs) exhibit an enhanced performance grade, a lower thermal susceptibility, a higher fatigue life, and a decreased accumulation of permanent deformations. Nonetheless, although GMABs stand out significantly from traditional alternatives, there is still no consensus on their behavior regarding chemical, rheological, microstructural, morphological, thermogravimetric, and surface topography properties. Therefore, this research conducted a literature review on the properties and advanced characterization techniques of GMABs. Thus, the laboratory protocols covered by this manuscript are atomic force microscopy, differential scanning calorimetry, dynamic shear rheometer, elemental analysis, Fourier transform infrared spectroscopy, Raman spectroscopy, scanning electron microscopy, thermogravimetric analysis, X-ray diffraction, and X-ray photoelectron spectroscopy. Consequently, the main contribution of this investigation to the state-of-the-art is the identification of the prominent trends and gaps in the current state of knowledge.

## 1. Introduction

Transportation infrastructure is a crucial element for the socioeconomic development of communities [1,2,3,4]. The preceding is evident in the growth of pavement construction and maintenance projects worldwide [5,6,7,8]. Notably, most of the road infrastructure in the world corresponds to asphalt pavements [4,9,10]. In this way, the demand for asphalt binders has soared at an accelerated rate. Consequently, the environmental impacts of this industry are immense and tend to grow over the years [11,12,13]. Therefore, researchers have proposed various strategies to decrease these environmental burdens [14,15,16]. One of the most promising alternatives is the production of high-performance asphalt binders [17,18,19]. The concept behind this approach states that employing materials with a longer useful life can mitigate the depletion of raw materials (i.e., non-renewable resources) and, thus, increase sustainability (both in environmental and economic criteria) in the long term [20,21,22].

Overall, high-performance asphalt binders are achieved by mixing the virgin/pristine binder with an additive agent [23,24,25,26]. Within these materials, the Graphene Modified Asphalt Binders (GMABs) stand out for their excellent mechanical behavior in a wide range of temperatures and frequencies [27,28,29,30]. The GMABs are the result of modifying the asphalt binder with graphene-like materials (i.e., graphene and its derivatives) [31,32,33,34]. Although there are many derivatives of graphene, only two are widely used in pavement engineering: Graphene Oxide (GO) and Graphene Nanoplatelets (GNPs) [35,36,37,38]. Remarkably, the flake graphite and expanded graphite nanosheets are other derivatives utilized to improve the engineering characteristics of asphalt binders [31,32,39].

In the last two decades, nanomaterials have been used with great notoriety to modify a broad type of materials, including asphalt binders [18,40,41,42]. The main nanomaterials employed for developing high-performance asphalt binders are chemical compounds based on carbon, for instance, graphene-like materials, carbon nanotubes, and nanoclays [43,44]. These modified asphalt binders are attractive because they (adequately) support high-traffic loads under harsh external conditions (i.e., high humidity, great environmental salinity, and intense ultraviolet radiation) at high and low temperatures [10,35,45]. Notably, the GMABs are one of the most versatile and resistant binders (i.e., provide an augmented service life) since they simultaneously improve fatigue and rutting resistances [31,32,36,46]. The preceding is particularly important because the asphalt mixtures (and successively the asphalt pavement structures) tend to fail mainly due to distresses associated with low fatigue life and raised accumulation of permanent deformations [18,38,40,47]. Another of the primary advantages of GMABs is that they are favorably compatible (to be blended) with other technologies, such as crumb rubber, electric arc furnace slag, epoxy resins, polystyrene, and Styrene-Butadiene-Styrene (SBS) [38,48,49,50,51,52].

Unlike neat asphalt binders, GMABs have such a complex chemical structure that they cannot be characterized only with traditional laboratory tests (i.e., density, penetration, softening point, dynamic viscosity, ductility, flash point, and solubility) [4,21,32,46,49]. Thus, in order to adequately describe the GMABs, it is necessary to carry out evaluations of their chemical, rheological, microstructural, morphological, thermogravimetric, and surface topography properties [34,53,54,55]. However, there is still no consensus in the literature on what particular tests should be conducted for these characterizations; moreover, there is much less consensus on the behavior of the GMABs under these laboratory protocols [31,32].

The preceding situation has motivated this manuscript to develop a comprehensive literature review on the properties and characterization techniques of GMABs. In this way, the main objective of this manuscript is to identify the main trends and gaps in the current state-of-the-art. Thus, it is expected that this investigation encourages researchers to improve their current practices and address those aspects that are still susceptible to improvement in the future. It is important to note that the scope of this review is limited to three graphene-like materials, i.e., graphene, GO, and GNPs.

Following, the structure of the subsequent sections of this paper is described. Section 2 presents the basics of the asphalt binder, emphasizing the aspects related to its chemistry. Next, Section 3 clarifies the essential concepts and features of graphene-like materials. In Section 4, the properties of GMABs are detailed together with the main modification processes. Likewise, Section 5 examines the state-of-the-art characterization techniques for GMABs, i.e., Atomic Force Microscopy (AFM), Differential Scanning Calorimetry (DSC), Dynamic Shear Rheometer (DSR), Elemental Analysis (EA), Fourier Transform Infrared Spectroscopy (FTIR), Raman Spectroscopy (RS), Scanning Electron Microscopy (SEM), Thermogravimetric Analysis (TGA), X-ray Diffraction (XRD), and X-ray Photoelectron Spectroscopy (XPS). Successively, in Section 6, there is a discussion about the trends and gaps in the literature. Then, Section 7 proposes recommendations for future research lines. Finally, Section 8 lists the main conclusions of this investigation.

## 2. Basics of Asphalt Binder

Asphalt binder is one of the derivatives obtained from refining crude oil (also named petroleum) [56,57,58,59,60,61,62]. Asphalt binder is a material with outstanding hydrophobicity and excellent adhesion capabilities, allowing it to be employed in a wide range of applications, for instance, as a binder for producing composite materials (e.g., asphalt mastic and asphalt mixtures) and as a coating membrane for waterproofing projects [9,63,64,65,66,67,68].

**Figure 1:**
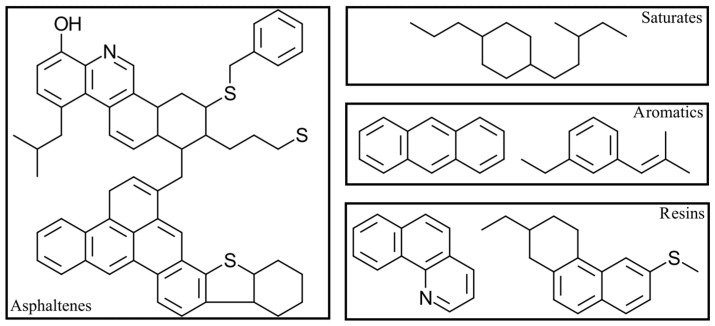
Standard molecular structure of the SARA fractions. Adapted from [3].

**Figure 2:**
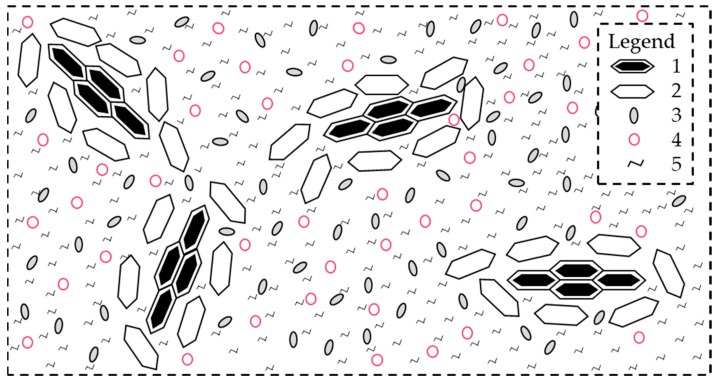
Colloidal model of the asphalt binder. Adapted from [3]. Legend: 1—central part of the asphaltene; 2—compounds with aromatic nature and high molecular weight; 3—compounds with prevalent aromatic nature and low molecular weight; 4—compounds of mixed aromatic–naphthenic nature; 5—compounds of mixed naphthenic–aliphatic nature.

Due to the variety of petroleum sources, each asphalt binder has a distinct chemical composition and a distinct performance for engineering applications [59,69]. Beyond these differences, asphalt binders can be chemically described as an intricate combination of thousands of hydrocarbon molecules, oxygen compounds, and small amounts of nitrogen and sulfur compounds with traces of metals [57,70,71,72,73,74,75,76]. Notably, a singular assortment of hydrocarbon and oxygen compounds generates a specific temperature-dependent viscoelastic behavior [67,77]. These compounds can be classified from lower to higher polarity as Saturates, Aromatics, Resins, and Asphaltenes, i.e., the so-called SARA fractions [74,78,79,80]. Figure 1 illustrates their standard molecular structure. Remarkably, the SARA fractions are a colloidal system in which micelles of asphaltenes remain in maltenes (i.e., aromatics, resins, and asphaltenes) [63,73]. In other words, the high polar solid particles are dispersed constantly in the low polar oily environment [81,82,83]. Figure 2 exhibits a sketch of this model.

The equilibrium of SARA fractions is essential in the behavior of asphalt binder because the maltenes allow the proper state of fluidity/workability, and the asphaltenes ensure adhesion with aggregates [63,84,85]. Some specific trends that asphalt binders have according to the distribution of their SARA fractions are listed below [3,63,72,78,86,87]:

- If the amount of resins and asphaltenes increases, the asphalt binder exhibits a more solid-like state. As a result, it causes an increase in stiffness.
- If the amount of saturates and aromatics increases, the asphalt binder exhibits a more liquid-like state. As a result, it causes a decrease in stiffness.
- If the amount of aromatics and asphaltenes increases, the asphalt binder reaches better properties at high temperatures.
- If the resin content increases, the viscosity of the asphalt binder augments proportionally.
- Adequate amounts of saturates, aromatics, and asphaltenes provide an appropriate fluid state.
- A low asphaltene content improves the temperature sensitivity behavior.

In this way, it is evident that a delicate chemical equilibrium in the SARA fractions widely controls the engineering performance of asphalt binders. The preceding is particularly important because incorporating graphene-like materials alters the SARA fractions. Thus, it explains why GMABs exhibit such complicated behavior, which is difficult to understand only by using traditional laboratory protocols. Subsequent sections of this manuscript detail the characteristics and properties of GMABs.

**Figure 3:**
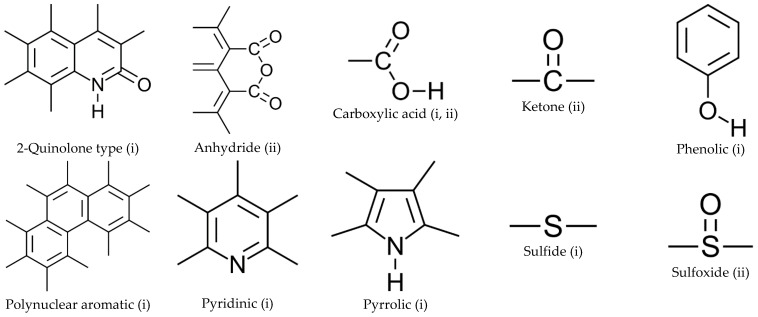
Functional groups formed during the oxidation aging mechanism. Adapted from [3]. **Legend**: (i) Naturally occurring; (ii) Induced by oxidation processes.

The further substantial effect that graphene-like materials have in asphalt binders is that the introduction of these additives prevents the oxidation aging mechanism (by extending the diffusion path of the gas) [88,89,90,91]. Notably, oxidation is the mechanism by which atmospheric oxygen diffuses throughout the asphalt matrix [63,92,93]. In turn, when the oxygen reaches the organic molecules of the asphalt binder, three chemical changes are assembled: fragmentation, oxygen addition, and condensation [92,94]. First, the large molecules are broken during the fragmentation, producing the smaller ones [92,95]; thus, the proportion of molecules with high molecular weight is progressively diminished [69,96]. Subsequently, the oxygen addition corresponds to constituting new functional groups, such as carbonyl (C=O), sulfoxide (S=O), hydroxyl, acid, and ester [77,79,92,97]. Figure 3 shows the chemical structure of some of these functional groups. Finally, during the condensation (also called the carbonization process), the aromatic (benzylic carbon group) and the larger, weightier molecules are formed due to polyaromatic oxidation reactions [63,92,98,99,100]. Consequently, the oxidation mechanism causes an increase in the stiffness of the asphalt binder and a loss of its adhesion/cohesion properties, which make it brittle, thus promoting cracking failure [101,102,103,104].

Consequently, it is notorious that the oxidation mechanism generates a complicated chain reaction of molecular rearrangements [3,105,106]. Furthermore, these reactions are complex when asphalt binders are modified with graphene-like materials [31,32]. Therefore, in order to correctly characterize the GMABs, it is necessary to perform chemical, rheological, microstructural, morphological, thermogravimetric, and surface topography tests [34,53,54,55].

## 3. Basics of Graphene-Like Materials

Despite being (correctly) isolated less than two decades ago (i.e., in 2004), graphene is a widespread substance with applications in dozens of heterogeneous industries (e.g., adsorption of pollutants, composite structures, detection of pesticides, combat against infectious diseases, lithium-ion battery manufacturing, membrane distillation, and wastewater treatment) [107,108,109,110,111,112,113,114,115]. Graphene can be defined as one of the allotropic forms of carbon, which has the shape of one thick atom layer of graphite [116,117,118]. Notably, the graphene’s carbon atoms are organized as a honeycomb lattice with a network of delocalized π electrons [119,120,121]. In this way, graphene is an inorganic 2D nanomaterial hexagonally arranged in a structure through an sp2 hybridization [112,122,123]. Normally, graphene can be found in three forms: fullerene, nanotube, and graphite sheets [32,107]. On the other hand, graphene has many derivatives (also called “graphene family nanomaterials”), such as expanded graphite nanosheets, flake graphite, fluorographene, nanographite, reduced graphene oxide, graphane, graphene nanocomposites, graphene nanoribbon, graphene nanosheets, graphene quantum dots, graphone, and graphyne [124,125,126,127]. However, the most used (in different sectors) are the GO and GNPs [31,37,39,128,129].

The GO is the 2D material formed by oxidizing graphite, which is usually achieved with chemicals, such as sulphuric acid [33,51,88,91]. GO stands out over graphene and graphite because it has large amounts of surface functional groups, such as hydroxyl, carboxylic, and epoxy [31,40,130]. Moreover, unlike graphene, GO exhibits excellent dispersion in the aqueous solutions [18,40,131]. The preceding permits its correct blend and compatibility with the asphalt binder and other additives [18,35,52,132]. For instance, GO can promote the absorption of aromatics and saturates from SBS polymer to enhance the temperature response of the GMABs [30,31,51].

GNPs are platelet-like graphite nanocrystals formed by several graphene layers (usually less than ten) [10,32,133,134]. On the one hand, GNPs stand out over pure graphene and other derivatives due to a low production cost (at least regarding large-scale fabrication analysis) [133,135,136]. Furthermore, the GNPs exhibit a morphological characteristic of narrow distribution, an ultra-high aspect ratio, a significantly lighter weight, an ultra-high aspect ratio, a tensile strength of 101 GPa, and Young’s modulus of 0.8–1 TPa [10,46,137]. Notably, GNPs develop an increased melting temperature and a low coefficient of thermal expansion that offers remarkable modifying-agent features [138,139,140,141]. Consequently, GNPs can be used at low concentrations to yield high-performance nanofluids [142,143,144].

Some main preparation methods for graphene-like materials are anodic bonding, chemical synthesis, using benzene as the building block, chemical vapor deposition process, growth from SiC, liquid phase exfoliation, micromechanical cleavage, molecular beam epitaxy, photoexfoliation, and precipitation from a carbon-containing metal substrate [29,32,145,146]. These preparation techniques can be classified as chemical preparation, physical preparation, micromechanical methods, and ultrasonic dispersion methods [32,34,39,147]. Regardless of the method employed for their preparation, the above nanomaterials present remarkable properties in common, i.e., elevated specific surface area, increased absorption, low bulk density, high functional density, great light transmittance, functional group-rich surfaces, and strong thermal-electrical conductivity [18,31,148]. Table 1 summarizes the main characteristics of these graphene-like materials. Due to these properties, graphene, GO, and GNPs have been used to modify asphalt binders [9,40]. In this way, the produced GMABs achieve a more extended service life with better performance (in comparison with a traditional unmodified asphalt binder) [31,32]. The subsequent section of this manuscript discusses the principal features of GMABs.

## 4. Properties of GMABs

Table 2 shows the effects of graphene-like materials on asphalt binder behavior. Overall, the GMABs present superior performance over traditional asphalt binders regarding fatigue and rutting resistances [31,32,90,158]. In this table, it is also evident that incorporating these nanomaterials causes the asphalt binder to increase its viscosity [18,147,155]. Because of this, GMABs used to require higher mixing temperatures (to produce asphalt mixtures) than conventional asphalt binders [39,50,88]. Likewise, the optimal binder content developed by GMABs is more elevated than the associated with neat asphalt binders [40,137,159,160].

GMABs have remarkable resistance to different aging processes, i.e., thermal, ultraviolet, and even water aging [21,30,89,90,161]. Nonetheless, the GMABs may exhibit increased volatilization of light components due to the high temperatures required for their production [3,162]. The preceding is associated with the large specific surface area of graphene-like materials, which makes it difficult to disperse in some fluid media [31,32]. Notably, in order to facilitate the dispersion of graphene-like materials within the asphalt matrix, it is feasible to incorporate solvent or dispersant agents [119,163]. For instance, some common solvents are trichloroethylene and anhydrous ethanol [9,39,91,163]. Meanwhile, the dipropylene glycol dimethyl ether and polyvinyl pyrrolidone are typical dispersants [31,39,155]. Although there is still no total consensus in the literature, it is found as a majority trend that GO disperses more easily than graphene and GNPs [31,119]; this may be associated with the enriched number of oxygen-containing functional groups that the GO has [132,164].

Although the effects described in Table 2 are widely accepted in the literature, it is essential to clarify that the physicochemical properties of GMABs depend on the preparation process used to disperse and blend the graphene-like materials within the asphalt matrix [34,89,163]. There are three paramount manners for preparing GMABs, namely the direct addition method, the indirect addition method, and the auxiliary addition method [29,31,147]. These methods are described below [31,32,33,146]:

- Direct addition method: the graphene-like materials are directly added into the asphalt binder (previously elevated to a high temperature).
- Indirect addition method: the graphene-like materials and asphalt binder are simultaneously dissolved into a medium solution to subsequently form a uniform solution.
- Auxiliary addition method: the graphene-like materials are first altered by specific functional groups, and then, the new modifying agent is melted into the asphalt binder.

The graphene like-materials exhibit a large shape ratio (i.e., diameter/thickness), increased specific surface area, and scalable pore dimension [17,145,147,165]. Due to these properties, the geometry of the graphene-like materials controls the performance of the GMABs [49,50]. Notably, as the particle size of the modifying agent decreases, the GMABs increase their free volume fraction, glass transition temperature, and shear viscosity [18,83,128,138]. In other words, the particle geometry controls the low-temperature behavior and resistance to the permanent deformation [27,28,155,166,167]. Table 3 presents several case studies regarding GMABs and their performance. This table also includes information on graphene-like materials’ particle geometry.

## 5. Characterization Techniques for GMABs

GMABs are usually initially characterized with a traditional test protocol, that is, the set of evaluations for density, penetration, softening point, dynamic viscosity, ductility, flash point, and solubility [4,10,18,21,32,46,49]. Nevertheless, these tests are insufficient to understand the thermo-dependent viscoelastic behavior of the GMABs [31,32]. For these purposes, it is necessary to resort to more advanced tests, such as AFM, DSC, DSR, EA, FTIR, RS, SEM, TGA, XRD, and XPS [4,21,32,49,169,170]; these are described below. In this way, it is possible to comprehensively assess chemical, rheological, microstructural, morphological, thermogravimetric, and surface topography properties. Moreover, these sophisticated tests allow to evaluate and ensure the correct dispersion of graphene-like materials within the structure of the asphalt matrix [119,158,171,172].

### 5.1. AFM

The AFM is a microscopy designed to record the topography of materials at a sub-nanometric scale for liquid and air media [173,174,175,176]. The AFM has been widely used to study GMABs [9,89,132]. The AFM employs a sharp-stylus probe to scan the material’s surface by exploring the repulsive and attractive forces between the material and the probe [177,178,179]. In this way, a high-resolution dimensional topographic image in 2D or 3D is formed [49,89,166]. The analysis of these images (usually conducted with specialized software/algorithms) can yield additional information (not exclusive to the surface topography), such as adhesion forces, elasticity, electrostatic force, morphology, nanoindentation, nano-phase separation, roughness, and even stiffness [174,180,181]. The combined assessment of these properties can be utilized to identify changes in the asphalt binders’ chemical structure [180,182,183]. For instance, through the identification of the three main phases of the asphalt binder morphology, i.e., catanaphase (bee structures), periphrases (dispersed phase), and paraphase (the matrix) [61,78,183,184,185]. Overall, the graphene-like materials can augment the number of “bee” structures in the structure of the asphalt binder but diminish their size [132,166].

### 5.2. DSR

The DSR is an apparatus utilized to measure the rheological response of some fluids under a wide range of temperatures, frequencies, and shear stress [19,186,187,188,189]. For instance, the DSR is typically used to examine the temperature-dependent viscoelastic behavior of asphalt binders, including GMBAs [166,188]. This device can record essential properties/parameters, such as complex modulus, complex viscosity, elastic modulus, phase angle, strain, stress, and viscous modulus [190,191]. Likewise, these results make it feasible to compute other vital parameters, such as zero shear viscosity, rutting factor, fatigue factor, non-recoverable compliance, and DSR function [192,193,194]. In the literature, it has been reported that the fatigue and rutting factors are especially sensitive to change after modifying the asphalt binder with graphene-like materials [46,137].

### 5.3. EA and XPS

The set of techniques employed to estimate the proportion of the chemical elements that contain a material is denominated EA [195,196,197,198]. Thus, the EA has commonly used to outline the chemical changes that an asphalt binder undergoes after its modification [67,199,200,201]. A wide range of devices and techniques exist to conduct an EA, even though XPS is the most utilized in the road infrastructure industry [202,203,204]. The preceding is because the XPS is efficient and versatile [3,205]. Overall, the XPS emits X-ray photons (with distinct energy) to excite electrons in the innermost orbitals of atoms [206,207]. Hence, a distinctive energy spectrum is generated, which contains peaks corresponding to the structure of the atoms found on the surface of the analyzed sample [204,208,209,210]. Consequently, it is easy to establish the chemical elements (and their proportions) that compose a material [211,212,213]. For instance, after modifying the asphalt binder with graphene-like materials, it is expected that the asphalt matrix will undergo an increase in the carbon ratio and a decrease in oxygen [53,214,215].

### 5.4. FTIR

The incorporation of graphene-like materials within the asphalt binder causes the asphalt matrix to alter the functional groups, i.e., some are transformed into new ones [9,37,169]. However, there is still no consensus regarding chemical reaction paths [89,130,146]. Notably, the chemistry of the GMABs will depend on the specific composition of the raw materials employed [29,34,161]. Therefore, in order to understand the changes suffered by the functional groups from a neat asphalt binder to a GMABs, it is necessary to carry out an FTIR analysis [28,90,166].

**Figure 4:**
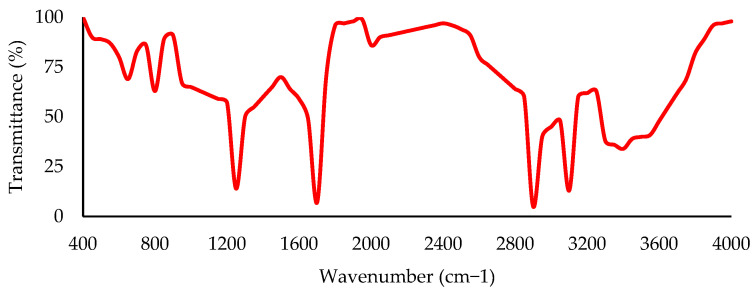
Typical graph of an FTIR analysis conducted in an asphalt binder sample. Adapted from [3].

FTIR is a spectrometry method to estimate the capacity of a material to absorb light (infrared radiation) regarding a characteristic wavelength range [57,216]. Therefore, the FTIR computes the spectral bands, which are the brusque changes in the transmittance vs. the wavenumber [71,217,218]. Figure 4 shows a sketch of a typical plot from an FTIR analysis. In this way, since each functional group has a specific spectral band, it is feasible to calculate the proportion of one [44,219,220]. Although there are several approaches to calculating it, the most popular way is to divide the area under the curve centered on the specific spectral band of a functional group into the area under the curve of the entire spectrum evaluated [161,190,221]. It is important to note that the FTIR has a central problem: there is no unanimity on the specific wavenumbers and the entire spectral band to be considered [3,216]. The preceding can be evidenced in Table 4. This table presents the wavenumbers and spectral bands used to compute the functional group indexes in some case studies for C=O, S=O, aromatic, and butadiene groups. Thus, the proper producibility and replicability of the results could be affected.

### 5.5. RS

As its name indicates, the RS is a spectroscopy technique that emits/relies upon Raman scattering (i.e., inelastic scattering of photons) in a specific (monochromatic) electromagnetic spectrum [230,231]. Usually, the RS devices detect and measure the vibration changes (scrolling up and down) in the system produced by the energy of the laser photons [232,233]. With this procedure, it is possible to deeply study a sample and, thus, obtain a “fingerprint” for each molecule [234,235]. In other words, RS allows the identification of the presence (in its quantity) of particular chemical compounds with high precision [236,237,238]. For the specific case of the GMABs, it is feasible to determine the content of graphene derivatives (i.e., carbonous materials) and their number of layers [169,239,240]. Consequently, the carbon molecules’ diffusion degree (or coupling) within the GMABs can be estimated [241,242]. Although there is no consensus on the spectral range to be employed with GMABs, it is typical to consider a range between 800–2000 cm^−1^ [146,169]. Undoubtedly, it is expected that the amount of carbon molecules will increase remarkably after modifying the asphalt binder with graphene-like materials [54,243].

### 5.6. SEM

The SEM is a method for scanning and analyzing the microscopic morphology of organic and inorganic materials [244,245]. The resolution of the SEM tests can vary from micrometer to nanometer scale [246,247]. Overall, the SEM applies backscattered and low-energy secondary electrons to estimate the changes in the topography of a material’s surface [248,249]. Therefore, it is feasible to generate 3D images exhibiting the microstructural characteristics of the sample [250,251]. Although the results of SEM tests on GMABs may vary depending on the modifying agent used, it is almost considered by consensus that graphene-like materials cause the asphalt binder to develop a multilayer stacked flake structure with smooth and flat surface similar to crystal stone-like grooves [9,90,155,167].

### 5.7. TGA and DSC

The GMABs can develop complex thermal stability, varying over time [43,166]. Although there is no consensus on this criterion, thermal stability can be assessed by employing a TGA [252,253]. The TGA is a method that evaluates sample mass changes during a gradual temperature increase (even up to 1600 °C) [254,255,256,257]. Therefore, this test permits estimating different parameters related to the activation energy, decomposition, reaction kinetics, resistance to pyrolysis, and thermal behavior [252,258,259,260]. For asphalt binders, it is typical to draw a curve of temperature augments vs. mass lost ratio and then look for abrupt or accelerated changes (low thermal stability is evidenced with very noticeable changes) [252,253,261]. Notably, there is a wide range of TGA protocols; however, the DSC is the most widely used for petroleum-derived products [180,262,263,264,265]. The DSC stands out because it is the only direct method that allows estimating the enthalpy of a process and also indicates correlations between the physical properties of substances with their thermal behaviors [266,267,268]. Remarkably, the glass transition temperature is the most advantageous parameter for examining asphalt binders utilizing the DSC analysis [27,37,52]. This temperature is the middle point where the glass transition appears [26,60].

### 5.8. XRD

After modifying an asphalt binder with graphene-like materials, the asphalt matrix develops high quantities of new crystal structures [9,49,158]. Accordingly, one way to measure the degree of coupling between the molecules of the asphalt binder with molecules of the modifying agent is to evaluate the formation of crystalline structures [42,162,269,270]. Moreover, adding graphene-like materials diminishes the oxygen- containing functional groups (especially on the structural layer of carbon atoms) [9,32,48]. The preceding can be conducted through the XRD technique [271,272,273]. An XRD device implements X-rays (i.e., high-energy electromagnetic energy with low wavelengths) to emit photons directly to a sample [274,275,276]. Then, the XRD measures and follows the diffraction patterns and peaks [146,241]. In this way, it is possible to obtain data c ng the atomic structure of a specific material [163,277]. Likewise, examining the position of atoms and their arrangement is also feasible [278,279]. Notably, because this is a non-destructive test, the XRD has been used in various industries to study sensitive materials [280,281].

### 5.9. Summary of Characterization Techniques

**Figure 5:**
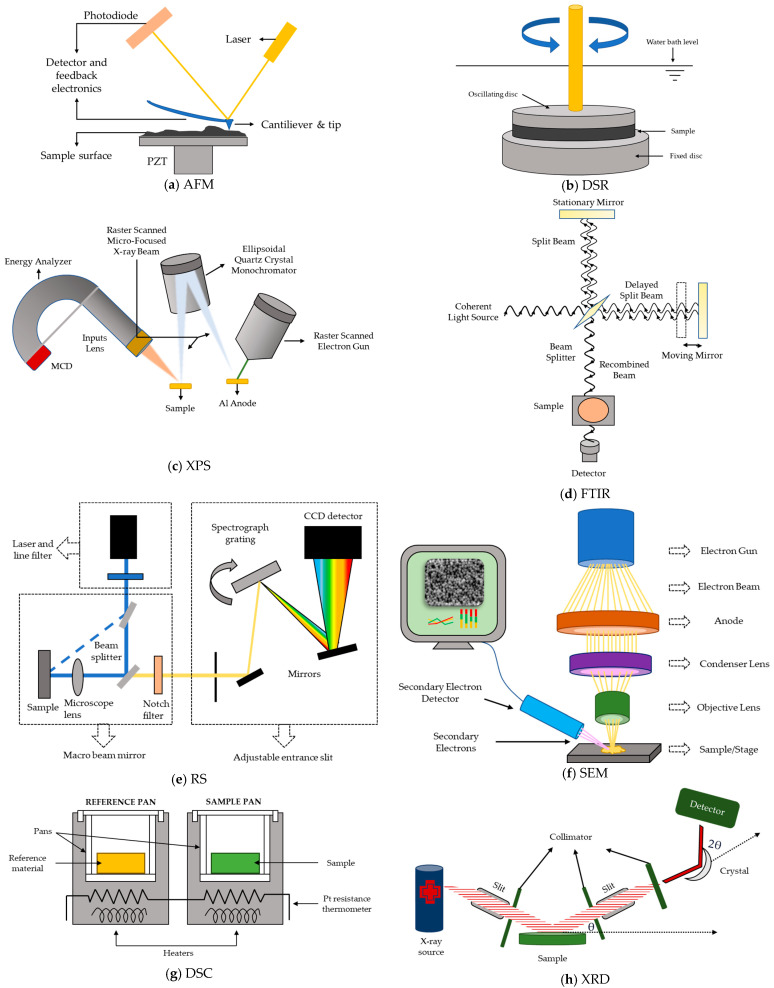
Schematic description of the functioning of the leading advanced characterization techniques for GMABs. Adapted from [285,286,287,288,289,290,291,292].

Table 5 summarizes several investigations in which the preceding characterization techniques were implemented to analyze asphalt binders. Specifically, this table presents the central findings of various case studies on GMABs. In this way, each graphene-like material’s main effects on the behavior and performance of the asphalt binders are exhibited. Additionally, Figure 5 shows a schematic explication of each of these devices.

## 6. Discussion

Current investigation trends show that the researchers focus on applying optical techniques to study GMABs. Although these techniques are extremely useful for performing the physical–chemical characterizations of different materials, these laboratory protocols also can present significant disturbances and errors in the result reports [293,294]. Notably, optical-type techniques (basically all varieties of microscopy and spectroscopy) employ light (i.e., photochemistry, luminescence, and light scattering) to probe or manipulate materials [295,296]. Unfortunately, small changes in the conditions (both external and internal) of the test can greatly influence the observations and measurements recorded [297,298]. Thus, disturbances or inconsistencies in the execution and post-processing of the tests may impair the reliability of the results [299,300]. Accordingly, the test outcomes depend on the sample preparation quality and the skills of the laboratory worker (i.e., control over the environment, mastery over the test apparatus, and the ability to interpret the results) [301,302]. Even though more of these aspects cannot be easily handled, there is one that can be, that is, the data post-processing [303,304]. Researchers’ main difficulty when using these optical techniques is interpreting the resultant images/photography manually [305,306]. Fortunately, this can be addressed by employing computational methods, such as artificial neural networks [245,250]. By implementing this type of artificial intelligence, it is feasible to analyze images in detail and find patterns, arrangements, and changes that could be effortlessly ignored (if they are done traditionally) [236,247]. Nonetheless, in the literature review, no research was found using machine learning to examine the test results on GMABs. The preceding represents an important gap in the literature.

On the other hand, another critical gap was evidenced in the literature: the tests on asphalt binders are usually conducted only before (i.e., on neat asphalt binders) and after (i.e., on GMABs) the modification processes. In this way, it is not common for the researchers to evaluate the properties of the GMABs over time, e.g., after several months of modifying the binder. The preceding implies that in the current state-of-the-art, there is no consensus on how the physical–chemical properties of the GMABs evolve in the long term. Consequently, this scenario is disturbing because the average service life of traditional asphalt pavements is 20 years, while it is 50 years in the case of perpetual pavements [12,307,308,309,310]. In other words, existing research trends do not permit knowing how GMABs would behave when used for road infrastructure construction. Notably, [55,90,161] evaluated the aging of the GMABs using the rolling thin film oven test, pressure-aging vessel test, and ultraviolet lamps. However, although these research efforts are not comparable to real-time long-term measurements [3,311], they demonstrate that some case studies have identified the need to carry out this type of assessment.

## 7. Future Research Lines

In light of the above, the current state-of-the-art about graphene-like materials and their applications to produce GMABs is susceptible to improvement. Therefore, some suggestions for future research lines are presented below: (i) implement machine learning to interpret the results obtained through advanced characterization techniques; (ii) research the physical–chemical change of the GMABs over the long term; (iii) develop mathematical–computational models to predict the effect of graphene-like materials on the asphalt binder performance; (iv) establish boundaries about the optimal geometric features that the graphene-like materials should have to guarantee the better possible behavior; and (v) evaluate the viability of other graphene derivatives, such as expanded graphite nanosheets, flake graphite, fluorographene, graphane, graphene nano-composites, graphene nanoribbon, graphene nanosheets, graphene quantum dots, graphone, graphyne, nanographite, and reduced graphene oxide.

## 8. Conclusions

In this research, a comprehensive literature review on the properties and characterization techniques of GMABs was carried out. Likewise, the main physicochemical aspects of asphalt binders and graphene-like materials were explored and discussed. In this way, it was possible to draw the following conclusions:

- Neat asphalt binders are modified with graphene-like materials to produce high-performance binders.
- The prominent advantages of GMABs over traditional asphalt binders are decreased thermal susceptibility and increased resistance to aging, fatigue, rutting, and moisture damage.
- The primary graphene-like materials are used to modify asphalt binders are graphene, GO, and GNPs.
- The main processes for producing GMABs are the direct addition method, indirect addition method, and auxiliary addition method.
- GMABs are highly compatible with a wide variety of additional modifying agents, such as carbon fibers, cross-linked chitosan, crumb rubber, electric arc furnace slag, epoxy resins, ethylene bis(stearamide), Fe^3+^-TA, glutaraldehyde, nickel, polyethylene, polystyrene, polyurethane, polyvinylpyrrolidone, and SBS.
- Conventional laboratory tests (i.e., density, penetration, softening point, dynamic viscosity, ductility, flash point, and solubility) are insufficient to characterize the complex behavior of GMABs. Therefore, it is necessary to employ advanced characterization techniques.
- In order to properly characterize the GMABs, it is essential to conduct assessments regarding chemical, rheological, microstructural, morphological, thermogravimetric, and surface topography properties. Notably, AFM, DSC, DSR, EA, FTIR, RS, SEM, TGA, XRD, and XPS are the leading tests for these purposes.
- There is still no consensus in the literature on the physicochemical properties of GMABs and their performance as a material for the road infrastructure industry. Regardless, all cutting-edge evaluation techniques indicate an improvement in low- and high-temperature performance (regarding the neat asphalt binders).
- Two primary gaps in the literature were identified: (i) although most of the advanced characterization techniques for GMABs are based on optical methods, researchers do not use computational approaches (such as artificial neural networks) to automatize the data interpretation process, and thus, reduce the inaccuracies associated with these observations/measurements; and (ii) not enough research efforts have been carried out to understand the behavior of the GMABs in the long-term.

## Figures and Tables

**Table 1:** Properties of main graphene-like materials.

Properties	Graphene	GO	GNPs	References
Appearance	Odorless black powder			[39,51,119]
Solubility in water	Negligible	Non-negligible		[119,149,150]
pH	8.3–11.4	Wildly variable		[119,151,152]
Number of layers	3–8	5–15	3–7	[18,35,40,51,133,153,154]
Diameter size, ⌀ (μm)	11–18	5–100	1–15	[18,35,40,51,119,133,153]
Thickness (nm)	1–2	1–8	2–3	[18,35,40,51,133,153]
Specific surface area (m^2^/g)	360	50–450	30–50	[18,35,39,40,51,153]
Bulk density (g/cm^3^)	0.4	0.9–1.8	0.01–0.13	[39,119,155]
C content (wt.%)	100	60–75	99.5	[40,88,119]
O content (wt.%)	-	20–35	Negligible	[88,156,157]
S content (wt.%)	-	2		
Mn content (wt.%)	-	1		
K content (wt.%)	-	1		
Si content (wt.%)	-	1		

**Table 2:** Effects of graphene-like materials on asphalt binder behavior. Adapted from [31,32,88,90,158].

Properties	Graphene	GO	GNPs
Aging resistance	↑	↑	↑
Density	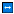	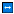	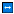
Ductility	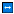	↓	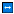
Fatigue resistance	↑	↑	↑
Flash point	↑	↑	↑
Loss modulus	↓	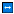	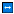
m value	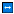	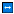	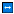
Moisture stability	↑	↑	↑
Penetration	↓	↓	↓
Recovery rate	↑	↑	↑
Rutting resistance	↑	↑	↑
Softening point	↑	↑	↑
Stifness modulus	↑	↑	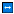
Storage modulus	↑	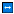	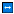
Thermal susceptibility	↓	↓	↓
Viscosity	↑	↑	↑

Legend: ↑ increment; ↓ reduction; 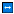 no explicit trend.

**Table 3:** Summary of case studies on GMABs.

References *	Neat Asphalt Binder **	Graphene-Like Material			Extra Modifying Agent
		Type	Geometry	Dosage (%)	
[9]	60/80 PNG	GO	⌀: 0.2–10 µm Thickness: 1–5 nm	0.06	Cross-linked chitosan, Glutaraldehyde, SBS
[10]	60/70 PNG	GNPs	⌀: 75 µm	0.5, 1, 1.5	-
[18]	60/70 PNG	GO	⌀: 10–50 µm Thickness: 1–1.77 nm	0.5, 1, 1.5, 2, 2.5	-
[27]	60/70 PNG	Graphene	⌀: <6 µm	2, 4, 6, 8, 10	-
[28]	60/70 PNG	GNPs	⌀: 5–10 µm Thickness: <3 nm	0.4	Polyethylene
[33]	60/70 PNG	GO	“not specified”	0.1, 0.3, 0.5, 0.7, 0.9	-
[52]	80/100 PNG	GO	⌀: 10–50 µm Thickness: 1 nm	0.2, 0.5, 1	Epoxy oligomer
[119]	PG 76–22	Graphene	⌀: 11 µm	0.3, 0.65, 1, 1.5, 2.5, 5, 7	SBS
[129]	60/80 PNG	GNPs	“not specified”	0.02, 0.08	Polystyrene, SBS
[130]	80/100 PNG	GO	“not specified”	0.5, 1	Polyurethane
[131]	70/90 PNG	GO	⌀: 15–20 µm	0.5, 1, 1.5, 2	-
[137]	60/70 PNG	GNPs	⌀: 2–7 µm Thickness: 2–10 nm	2, 4	-
[147]	60/70 PNG	GO	Thickness: 50–80 nm	0.2, 0.4, 0.8, 1.6	Polyurethane
[155]	60/80 PNG	GNPs	⌀: 1–15 µm Thickness: 2.4 nm	0.5, 1, 1.5, 2	Polyvinylpyrrolidone, SBS
[158]	~80 PNG	Graphene	⌀: 5–50 µm Thickness: 3.4–8 nm	0.5, 1, 1.5	-
[163]	PG 64-22	Graphene	⌀: 7–15 µm	2–20	Ethylene bis(stearamide)
[165]	60/80 PNG	Graphene	⌀: 5–50 µm Thickness: 3.4–8 nm	2, 4, 5.9	Carbon fibers, Nickel
[166]	80/100 PNG	GNPs	⌀: 5–50 µm Thickness: 3.4–8 nm	0.5, 1, 1.5, 2	SBS
[168]	60/80 PNG	GO	⌀: 0.2–10 µm Thickness: 1–5 nm	0.02, 0.04, 0.06, 0.08	Fe^3+^-TA, SBS

* In all case studies, a general improvement of mechanical properties was reported. ** PNG: penetration grade; PG: performance grade.

**Table 4:** Wavenumbers and spectral bands used to compute the functional group indexes. Adapted from [3].

Functional Group	Distinctive Wavenumber (cm^−1^)	Total Spectral Band Evaluated (cm^−1^)	References
C=O	around 1600	600 to 2000	[222]
	around 1694	600 to 2000	[44]
	around 1700	725 to 3570	[223]
	around 1702	722 to 2920	[218]
S=O	around 1015	725 to 3570	[223]
	around 1027	600 to 2000	[44]
	around 1030	500 to 4000	[224]
	around 1032	723 to 2924	[71,217]
Aromatic	around 1600	723 to 2924	[220]
	around 1601	600 to 2000	[60,225]
	around 1601	723 to 2924	[71,217]
	around 1606	725 to 3570	[223]
Butadiene	around 966	600 to 2000	[222,226,227]
	around 966	722 to 2920	[218]
	around 968	600 to 2000	[228]
	around 985	600 to 2000	[229]

**Table 5:** Summary of case studies on the analysis of GMABs with advanced characterization techniques.

Characterization Technique	Modifying Agent	Neat Asphalt Binder	Main Findings	References
AFM	Graphene	60/80 PNG	The graphene promotes the nucleation of bee structures, which augments the number and reduces the volume of these structures.	[55]
	GO	40/50 PNG	The GO causes a grafting reaction that yields wavy bends with a wide degree of curling.	[89]
	GNPs	60/80 PNG	THE AFM force curve analysis shows that GNPs reduce temperature sensibility and enhance plasticity and viscosity behavior.	[49]
DSR	Graphene	60/70 PNG	As the graphene content increases, the asphalt binder increases its viscosity and reduces its high-temperature susceptibility.	[27]
	GO	60/70 PNG	GO can greatly elevate the permanent deformation resistance of asphalt binders in a wide range of temperatures.	[35]
	GNPs	60/80 PNG	The GNPs inclusion enhances the high-temperature rutting resistance performance and the fatigue resistance.	[49]
EA and XPS	Graphene	40/50 PNG	The GMABs show three distinctive functional groups: nonoxygenated C-C (285.08 ev), ether C-0 (286.43 eV), and C=N bond (280 ev).	[282]
	GO	40/50 PNG	The strongest peaks caused by the GO were at 284.79, 286.61, 287.28, and 288.86 eV, representing C–C, C–O–Si, C–SH, and HO–C=O, respectively.	[89]
	GNPs	40/50 PNG	Regarding the GO, GNPs reduce the interplanar spacing of modified asphalt binder by approximately 48%.	[89]
FTIR	Graphene	40/60 PNG	The graphene yields anti-aging properties to the asphalt binder, at least in the spectral range of 1760–1500 cm^−1^.	[169]
	GO	40/50 PNG	The more substantial absorption peaks were at 3386.6 and 1375.7 cm^−1^, representing the hydroxyl group stretching and bending oscillation.	[89]
	GNPs	60/80 PNG	The GNPs modification was highlighted by characteristic absorption peaks at 1184.44, 1601.02, 1492.61, 1450.95, 755.12, and 698.43 cm^−1^.	[49]
RS	Graphene	40/60 PNG	The G-band (1580 cm^−1^) and the D-band (1350 cm^−1^) can assess the presence of graphene.	[169]
	GO	70/100 PNG	GO modification is characterized by distinctive D and G peaks at 1352 cm^−1^ and 1600 cm^−1^, respectively.	[17]
	GNPs	PG 58-28	The modification with GNPs provokes the prominent peaks: D-peak (1330 cm^−1^), G-peak (1580 cm^−1^), and 2D-peak (2660 cm^−1^).	[283]
SEM	Graphene	40/50 PNG	The graphene provokes a microcrack propagation path, easily distinguished through this technique.	[282]
	GO	40/50 PNG	The GO yields a clear sheet structure with a smooth surface.	[89]
	GNPs	60/80 PNG	GNPs develop notorious small wrinkles and ellipsoidal structures.	[49]
TGA and DSC	Graphene	40/60 PNG	The presence of graphene causes the asphalt binder to increase its maximum degradation temperature and melting peaks.	[169]
	GO	60/80 PNG	GO can considerably enhance the thermal stability of asphalt binders.	[168]
	GNPs	PG 52-34	The modified asphalt binder reduces the susceptibility to moisture damage and increases the stiffness and resistance to failure conditions.	[284]
XRD	Graphene	60/80 PNG	Graphene develops a multilayered morphology with a peak centred at approximately 24°.	[158]
	GO	40/50 PNG	The GO generates a grafting process, which is easily identified by an interplanar spacing of 0.421 nm.	[89]
	GNPs	60/80 PNG	XRD pattern analysis demonstrates that the asphalt binder molecules increased the interlayer distance of GNPs.	[155]

## Data Availability

Data is contained within this article.
